# 652. Foodborne outbreak investigation at a wedding event – Batken, Kyrgyzstan, June 2022

**DOI:** 10.1093/ofid/ofad500.715

**Published:** 2023-11-27

**Authors:** Timur Dautov, Nasyat Kemelbek, Marina Malisheva, Dinagul Otorbaeva, Roberta Horth, Dilyara Nabirova

**Affiliations:** Central Asia Field Epidemiology Training Program, Gulcha, Osh, Kyrgyzstan; Central Asia Field Epidemiology Training Program, Gulcha, Osh, Kyrgyzstan; Central Asia Field Epidemiology Training Program, Gulcha, Osh, Kyrgyzstan; Central Asia Field Epidemiology Training Program, Gulcha, Osh, Kyrgyzstan; US Centers for Disease Control and Prevention, Dulles, Virginia; CDC Central Asia office, Almaty, Almaty, Kazakhstan

## Abstract

**Background:**

On August 2nd, 2022, local health departments were informed that over a hundred people had sought care or reported gastrointestinal illness after attending the same wedding event in Batken, Kyrgyzstan, on July 30th. We investigated to identify the source and associated risk factors to stop transmission.

**Methods:**

Using a retrospective cohort study design, we interviewed consenting wedding participants. A case was anyone who was linked to the event who became acutely ill or sought medical care with food poisoning symptoms. We searched public health surveillance records for additional cases who sought healthcare for suspected foodborne disease, a reportable illness, from July 30^th^ to August 2^nd^. Food and environmental samples were collected and tested. We used logistic regression to determine associations with case status.

**Results:**

Of 250 attendees, 201 consented; 110 were cases and 91 non-cases. Among cases, 8%were < 20 years old, 27% were >61 years old, and 80% were female. Top symptoms were fatigue (96%), abdominal pain (95%), diarrhea (95%), and fever (84%). Most cases developed symptoms < 24 hours of the event and 79% sought care. Odds ratio was 7.8 for egg salad containing chicken (95% confidence interval [CI]: 4.1-14.4, p< 0.01), 5.1 for grilled chicken (CI: 2.8-9.4, p< 0.01) and 3.9 for chicken salad (CI: 2.2-7.1, p< 0.01). Attack rate was 66% (105/160) among people who ate any chicken (95% of cases and 60% of non-cases). Patients were not tested. *Proteus vulgaris*, *Klebsiella spp*., and *Escherichia coli* were detected in chicken samples. *E. coli* was detected in 3/32 environmental samples.

**Conclusion:**

Contaminated chicken was the likely source of illness. Source pathogen is uncertain because patients were not tested. Symptoms and onset were more consistent with *Klebsiella spp*. than *E. coli*, but *Klebsiella spp* food-associated outbreaks are rare. Recommendations were made to ensure restaurant compliance with food safety measures. Diagnostic testing for gastrointestinal illness needs strengthening.

**Disclosures:**

**All Authors**: No reported disclosures

